# CSNK2A1‐mediated phosphorylation of HMGA2 modulates cisplatin resistance in cervical cancer

**DOI:** 10.1002/2211-5463.13228

**Published:** 2021-07-12

**Authors:** Zhan Shi, Ding Wu, Hao Xu, Ju Yang, Xiaoqing Sun

**Affiliations:** ^1^ Translational Medicine Center Shanghai General Hospital Shanghai Jiao Tong University School of Medicine Shanghai China; ^2^ Department of Urology Jinling Hospital Nanjing Medical University Nanjing China; ^3^ Department of Urology Huangshi Central Hospital Affiliated Hospital of Hubei Polytechnic University Edong Healthcare Group Huangshi China; ^4^ The Comprehensive Cancer Centre of Drum Tower Hospital Medical School of Nanjing University Clinical Cancer Institute of Nanjing University Nanjing China; ^5^ Department of Urology Affiliated Hospital of Xuzhou Medical University Xuzhou China

**Keywords:** cervical cancer, chemoresistance, cisplatin, CSNK2A1, HMGA2

## Abstract

The development of chemoresistance reduces the efficacy of anti‐cancer drugs. Cervical cancer is still one of the most common cancer types in developing countries. The oncogenic protein high mobility group AT‐hook 2 (HMGA2) is involved in the development and progression of tumors, although its role in chemoresistance of cervical cancer remains unclear. Here, we report that HMGA2 is highly expressed in cervical cancer and negatively correlated with cisplatin‐induced cell death. We performed liquid chromatography‐tandem mass spectrometry to demonstrate that HMGA2 has high potential to interact with casein kinase II A1 (CSNK2A1). Moreover, we observed that HMGA2 co‐localizes with CSNK2A1 in the nucleus by immunofluorescence. Binding of HMGA2‐CSNK2A1 was detected by immunoprecipitation assays. In addition, we identified that cisplatin induces an interaction between CSNK2A1 and HMGA2, thereby promoting the phosphorylation of HMGA2. CX‐4945, a CSNK2A1 inhibitor, could inhibit the phosphorylation of HMGA2 and sensitize tumor cells to cisplatin. Our results reveal that CSNK2A1‐dependent HMGA2 phosphorylation may partially underlie cisplatin‐resistance in cervical cancer, suggesting that HMGA2 phosphorylation may have potential as a predicative biomarker and therapeutic target to improve chemotherapeutic efficacy.

## Graphical abstract

The oncogenic protein high mobility group AT‐hook 2 (HMGA2) participates in the development and progression of tumors. Cisplatin may enhance the interaction between HMGA2 and serine‐threonine kinase casein kinase II A1, thereby causing enhancement of HMGA2 phosphorylation in a time‐ and dose‐dependent manner, which in turn affects the bcl‐2/bax pathway and the resistance of cervical cancer cells to cisplatin.

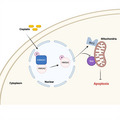

**Abbreviations**
CCK‐8: cell counting kit‐8
CSNK2A1: casein kinase II A1
DAPI: 4′,6‐diamidino‐2‐phenylindole
DDP: diamminedichloroplatinum
HMGA2: high mobility group AT‐hook 2
qRT‐PCR: quantitative real‐time polymerase chain reaction

Cervical cancer is the fourth most common cancer in women around the world, with an estimated half a million women diagnosed annually, and more than 300 000 women having died from this disease [1]. Although various therapies have been applied to treat cervical cancer, such as surgery, chemotherapy and radiotherapy, the prognosis is still poor [2]. One of the predominant reasons is chemoresistance. Therefore, it is necessary to exploit the underlying mechanisms in the development of chemoresistance in cervical cancer.

Cisplatin, an extensively employed chemotherapy agent in clinical practice [3], exerts anticancer activity by arresting DNA replication, causing DNA damage, blocking the production of genetic materials and inducing mitochondrial apoptosis [4, 5]. The resistance to cisplatin has turned out to be a predominant obstacle for its clinical application [6]. Therefore, exploring the mechanisms and pathways underlying cisplatin resistance might supply potential strategies to improve the clinical efficacy for cervical cancer.

As an architectural transcriptional factor, the oncogenic protein high mobility group AT‐hook 2 (HMGA2) is highly expressed in the early embryo and malignant tumours, such as prostate cancer [7], as well as esophageal squamous cell carcinoma [8], whereas it is absent or markedly diminished in normal adult tissues [9, 10]. HMGA2 increases cancer cell proliferation by promoting cell cycle entry and inhibiting cell death [11]. In addition, HMGA2 maintains the cancer stem cell phenotype and chemoresistance in breast cancer [12]. However, the underlying mechanisms of HMGA2‐mediated cisplatin resistance for cervical cancer remain largely unexplored.

Casein kinase II (CK2) is a highly conserved serine‐threonine kinase, it phosphorylates hundreds of cellular proteins and participates in the regulation of immunity, lipid metabolism and transcriptional elongation [13, 14, 15, 16]. It is composed of two catalytic (CK2α and CK2α′, which are encoded by the *CSNK2A1* and *CSNK2A2* genes, respectively) and two regulatory (CK2β, is encoded by the *CSNK2B* gene) subunits [17]. Of these, CSNK2A1 has been indicated to be involved in tumorigenesis of different malignancies. It is related to programmed cell death and autophagy via mediating the phosphorylation of specific proteins [18, 19]. CSNK2A1 has been considered as a prognostic and therapeutic target in a range of tumors. However, the role of HMGA2 and CSNK2A1 in cervical cancer needs to be deciphered.

To address this gap, we investigated the expression of HMGA2 in cervical cancer tissues and cell lines, and explored the role and potential mechanisms of HMGA2 in cisplatin resistance. Here, we report that the phosphorylation level of HMGA2 was associated with the chemoresistance in cervical cancer cells. Cisplatin could increase the phosphorylation of HMGA2 by enhancing the interaction between CSNK2A1 and HMGA2. Our findings support the potential role of HMGA2 as a novel target for cisplatin resistance and suggest the feasibility of combining cisplatin and HMGA2 inhibition for the improved chemotherapy of cervical cancer.

## Materials and methods

### Cell culture and materials

HEK‐293T (293T) cells, human normal cervical epithelial cell line (H8), human cervical cancer cell lines (HeLa, C33A and SiHa) and cisplatin‐resistant cell line HeLa/diamminedichloroplatinum (DDP) were purchased from the Chinese Academy of Science (Shanghai, China). All cells were grown in Dulbecco’s modified Eagle’s medium supplemented with 10% (v/v) fetal bovine serum in a humidified atmosphere of 5% CO_2_ at 37 °C. The anti‐Flag M2 agarose and monoclonal mouse antibody against Flag were obtained from Sigma‐Aldrich (St Louis, MO, USA). The polyclonal rabbit antibody against HMGA2 was purchased from the Abcam (Cambridge, MA, USA). The polyclonal rabbit antibodies against GFP, CSNK2A1, Bcl‐2, Bax and β‐actin were purchased from the Proteintech (Wuhan, China). Phospho‐Ser/Thr antibody was acquired from Cell Signaling Technology (Beverly, MA, USA). Cisplatin was obtained from MedChemExpress (Monmouth Junction, NJ, USA). The *Escherichia coli* strain BL21 (DE3) was purchased from TransGen (Beijing, China). Glutathione Sepharose 4B was purchased from GE Healthcare (Princeton, NJ, USA). CX‐4945 (small‐molecule CK2 inhibitor) was purchased from Selleckchem (Houston, TX, USA).

### Construction of stable cell lines

The plasmid containing HMGA2 was obtained by cloning the full coding sequences for the wild‐type into the vector of pcDNA3.0 vector or pLVX‐IRES, then confirmed by sequencing. The pLVX‐IRES‐HMGA2 was co‐transfected with the virus packing particles (PMD2.G and psPAX2) into the 293T cells for 3 days, then collected and the supernatants containing lentivirus were concentrated. Next, the HeLa cells were infected with the supernatants. After 48 h, we added the hygromycin to the infected cells, which were subsequently cultured for another 2 weeks. Next, the overexpression of HMGA2 was validated in the survived cells by a western blot assay. The HMGA2 knockdown stable cell lines were obtained as described previously [20]. The sequences of the HMGA2 knockdown double‐stranded oligonucleotides were: 5′‐CCGGAGTCCCTCTAAAGCAGCTCAACTCGAGTTGAGCTGCTTTGAGGGACTTTTTTG‐3′ (HMGA2‐sh1), 5′‐CCGGAGTCCCTCTAAAGCAGCTCAACTCGAGTTGAGCTGCTTTAGAGGGACTTTTTT‐3′ (HMGA2‐sh2). The packaged pLKO.1‐HMGA2‐shRNA was used to establish the stable HMGA2 knockdown cell lines.

### Quantitative real‐time PCR (qRT‐PCR)

We first extracted the total RNAs with the TRIzol reagent (Invitrogen, Carlsbad, CA, USA), then synthesized the complementary DNA using a cDNA Synthesis Kit (Vazyme, Nanjing, China) in accordance with the manufacturer’s instructions. The gene for HMGA2 was amplified using the primers 5′‐CAGGAAGCAGCAGCAAGAAC‐3′ (forward) and 5′‐GCCTCTTGGCCGTTTTTCTC‐3′ (reverse), the endogenous gene for GAPDH was amplified using the primers 5′‐GAAGGTCGGAG‐ TCAACGGATT‐3′ (forward) and 5′‐GAAGGGGTCATTGATGGCAAC‐3′ (reverse). The operation process, reaction conditions and the interpretation of the results matched those described previously [21].

### Western blot assay

At different time points, cells were harvested and treated with the lysis buffer containing the protease inhibitor and then centrifuged. The concentration was detected in accordance with the manufacturer’s instructions (Beyotime Bio‐technology, Nantong, China). Then, we used SDS/PAGE to separate the proteins and transferred them to the poly(vinylidene difluoride) membranes. The target proteins were detected and visualized using specific primary antibodies and appropriate secondary antibodies.

### Immunohistochemical analysis

The ethics committee of Shanghai General Hospital approved our study. Written informed consent was obtained from each of the patients who provided cervical cancer tissues. The study methodologies conformed to the guidelines set by the Declaration of Helsinki. The expression of HMGA2 in peri‐tumor and tumor tissues was measured by an immunohistochemistry assay as described previously [22]. Human tissue microarrays of cervical cancer (Superbiotek Pharmaceutical Technology, Shanghai, China) were purchased. The clinical characteristics of all samples were downloaded from the web sites of the apprpriate companies. Antibody against HMGA2 was used for immunohistochemistry staining. The intensity of HMGA2 staining was quantified, scored and graded (low, 0–4 points; medium, 5–8 points; high, 9–12 points).

### Cell proliferation assay

Cell proliferation was examined using a cell counting kit‐8 assay (CCK‐8) (Beyotime, Nantong, China). After treatment with cisplatin, cells were seeded into the 96‐well plates and then incubated for 24, 48 and 72 h, respectively. At different time points, the culture medium was removed and changed to Dulbecmodified Eagle’s medium with CCK‐8 solutions, followed by culturing for 1 h. The optical density in each well was measured at 450 nm via a spectrometer.

### Cell apoptosis assay

The stable cell lines of HMGA2‐overexpression or HMGA2‐knockdown and the control cells were treated with cisplatin, and then the cell death rates were detected using an annexin V‐fluorescein isothiocyanate/propidium iodide apoptosis detection kit (KeyGen, Nanjing, China) as described previously [7].

### Immunoprecipitation assay

After being transfected with the plasmids and treated with cisplatin for different time points, cells were harvested, lysed and centrifuged, then part of the supernatant was selected as the lysis part, whereas others were immunoprecipitated with anti‐Flag M2‐agarose overnight at 4 °C, then centrifuged, washed and blended with the loading buffer. TRhe sample was used for IP part. Protein samples were then analyzed by a western blot assay.

### *In vitro* phosphorylation assay

An *in vitro* phosphorylation assay was conducted as described previously [23]. Briefly, Flag‐CSNK2A1 was transfected into 293T cells for 24 h, and cells were treated with cisplatin for different times or doses before harvest. GST‐HMGA2 proteins were expressed in *E. coli* BL21 (DE3) and purified using Glutathione‐Sepharose 4B. Flag‐CSNK2A1 and GST‐HMGA2 complex were incubated at 30 °C in kinase buffer for half an hour. The phosphorylation of HMGA2 was analyzed by a western blot assay using anti‐p‐Ser/Thr antibody.

### Immunofluorescence *s*taining

Cells were seeded on the coverslips in 24‐well plates and transfected with plasmids if needed. After pretreatment as required, cells were fixed with 4% paraformaldehyde, permeabilized with 0.2% Triton X‐100, blocked with 2% bovine serum albumin, incubated with the primary antibodies, then stained with Alexa Fluor 488‐ or 568‐conjugated secondary antibodies. 4′,6‐diamidino‐2‐phenylindole (DAPI) was used for nuclear staining. Immunofluorescence was observed via fluorescence microscopy.

### Statistical analysis

All experiments were repeated at least three times. Data are shown as the mean ± SD, and processed with spss, version 20.0 (IBM Corp., Armonk, NY, USA). Statistical significance was performed using Student's *t*‐test. *P* < 0.05 was considered statistically significant.

## Results

### HMGA2 was highly expressed in cervical cancer

**Fig. 1:**
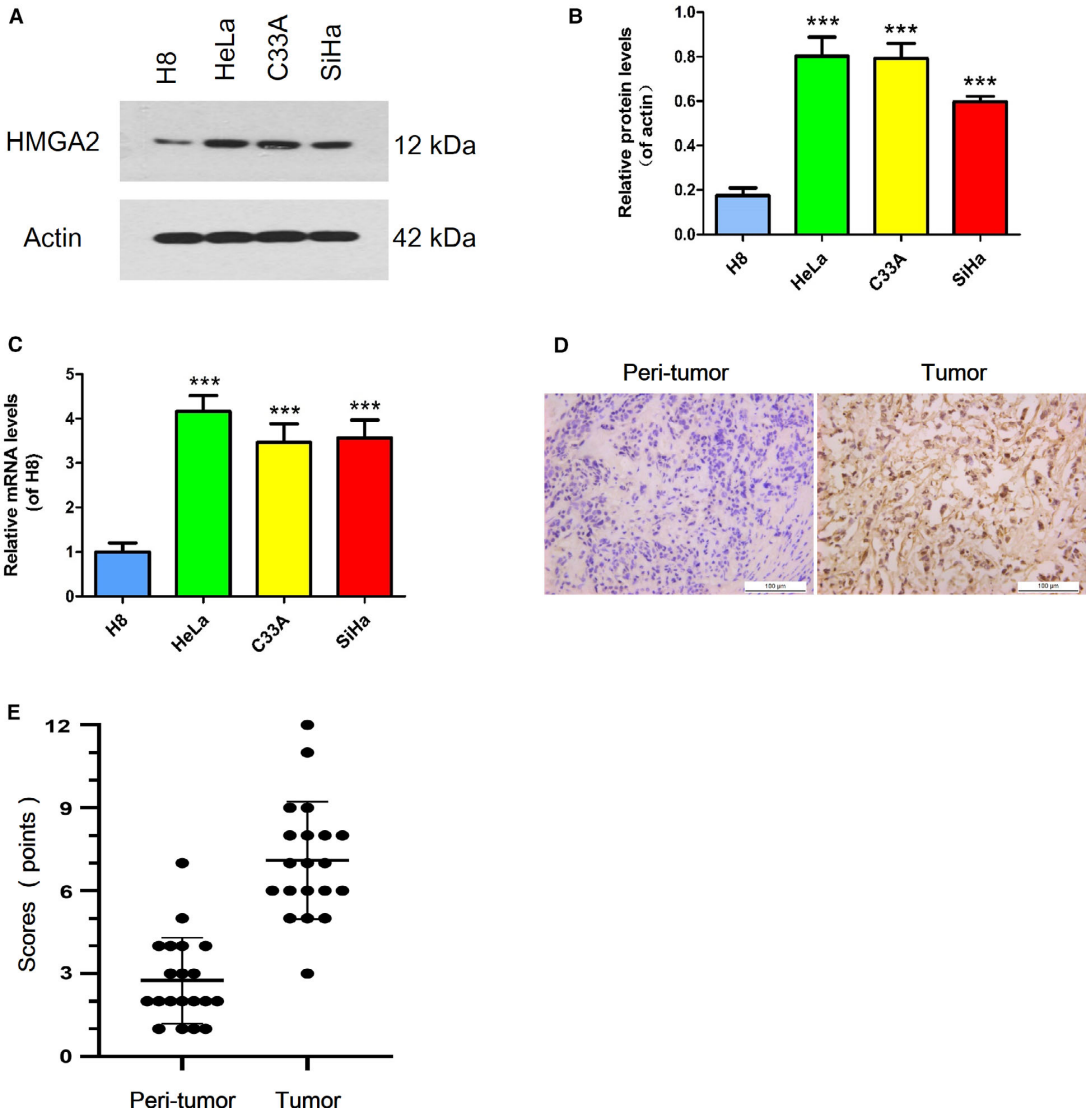
Expression of HMGA2 in cervical cancer. (A) Protein levels of HMGA2 were examined by a western blot assay in human cervical cancer cell lines and a normal cell line, with actin as the internal control. (B) Protein levels were quantified using imagej (NIH, Bethesda, MD, USA). (C) HMGA2 mRNA expression levels were tested by a qRT‐PCR assay and GAPDH expression was used for normalization. (D, E) HMGA2 protein expression was measured by immunohistochemical staining and quantified (scale bar = 100 μm). Data were analysed using Student's *t*‐test and are presented as the mean ± SD of three independent experiments. ***P < 0.001.

The function of HMGA2 in cervical cancer is unclear. Based on our previous study of prostate cancer, we detected the expression of HMGA2 in different cervical cancer cell lines and the normal cell line. As shown in Fig. 1A,B, we found that the protein levels of HMGA2 in cancer cells were higher than in normal cells, especially in HeLa cells, which were used for further experiments. Similar results were observed for HMGA2 mRNA expression as detected by a qRT‐PCR assay (Fig. 1C). Furthermore, HMGA2 protein levels were tested by immunohistochemical staining and found to be highly expressed in cervical cancer tissues compared to normal tissues (Fig. 1D,E). These results indicated that HMGA2 might correlate with the progression of cervical cancer.

### HMGA2 regulated cisplatin resistance in cervical cancer

**Fig. 2:**
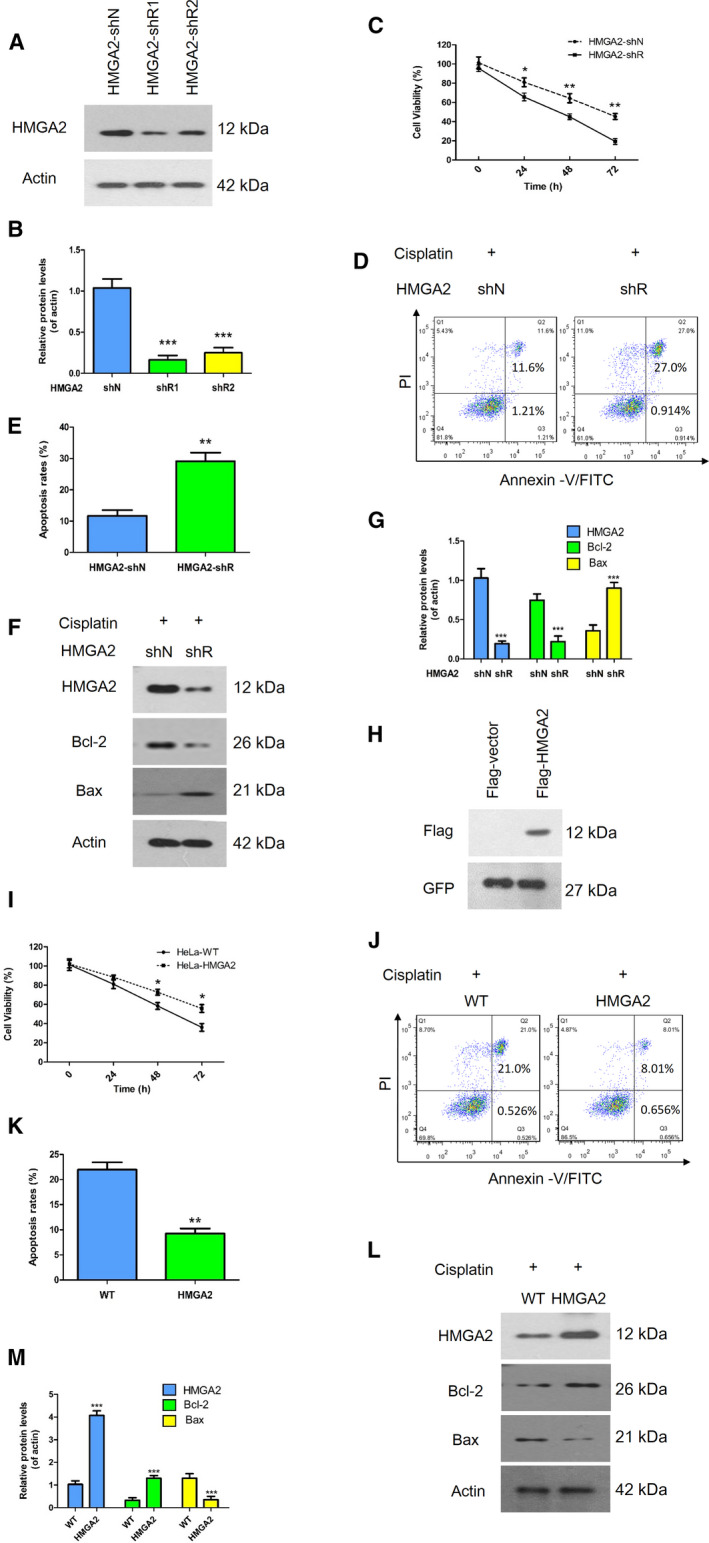
Effect of HMGA2 expression on cell viability during cisplatin treatment. (A, B) HMGA2 expression in HMGA2‐shN and HMGA2‐shR was examined by a western blot assay and quantified using imagej. (C) Knockdown of HMGA2 significantly inhibited cell proliferation compared to the controls. (D, E) HeLa cell apoptosis was detected voia flow cytometry using annexin V‐fluorescein isothiocyanate and propidium iodide. (F, G) The levels of several critical apoptosis markers were examined by a western blot assay. (H) Confirmation of the plasmid of Flag‐HMGA2. (I) A CCK‐8 assay was used to test cell viability with the overexpression of HMGA2 after treatment with cisplatin. (J, K) A representative image and quantified results show that cell apoptotic rates were decreased with HMGA2 overexpression. (L, M) A western blot assay was used to determine the expression of apoptosis‐related proteins. Data were analysed using Student's *t*‐test and are presented as the mean ± SD of three independent experiments, *P < 0.05, **P < 0.01 and ***P < 0.001.

To evaluate whether HMGA2 has an impact on the chemoresistance of cervical cancer, we established stable HMGA2 knockdown cell lines with the specific plasmids and evaluated the HMGA2 expression by a western blot assay (Fig. 2A,B). Then, we compared cell proliferation (by a CCK‐8 assay) and cell death (by a cell apoptosis assay) between HMGA2 overexpressing cell lines and HMGA2 knockdown lines. As shown in Fig. 2C–E, inhibition of HMGA2 resulted in lower cell viability and higher cell apoptosis rates after treatment with cisplatin. Given that Bcl‐2 and Bax are involved in the apoptosis pathway, we then detected the expression of Bcl‐2 and Bax in HMGA2 knockdown cells treated with cisplatin and found higher Bax but lower Bcl‐2 levels in HMGA2 knockdown cells compared to control cells (Fig. 2F,G), which was opposite to that found in HMGA2 overexpressing cells (Fig. 2H–M). These data suggest that HMGA2 might enhance cisplatin resistance for cervical cancer cells, although the underlying mechanism is not known.

### Cisplatin induced phosphorylation of HMGA2

**Fig. 3:**
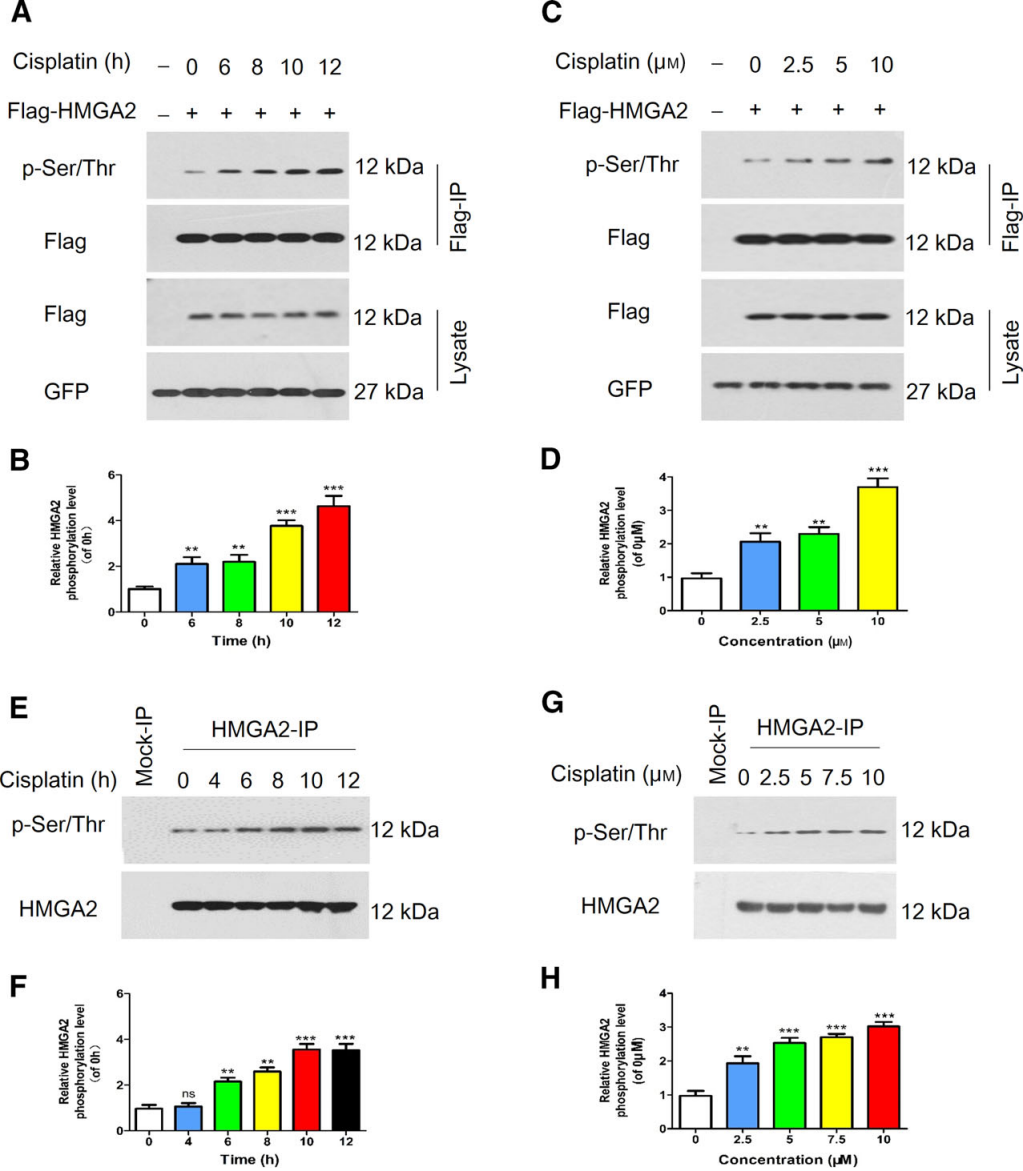
Effect of cisplatin on the phosphorylation of HMGA2. (A) 293T cells overexpressing Flag‐tagged HMGA2 were constructed and treated with cisplatin at different time points. Immunoprecipitation with an anti‐Flag antibody was performed. (C) After treatment with the indicated cisplatin concentration, 293T cells were harvested and immunoprecipitated to obtain immunoblots with the indicated antibodies. HeLa cells were cultured with cisplatin at various time points (E) or doses (G), then cell lysates were immunoprecipitated for HMGA2 and subjected to a western blot assay with anti‐HMGA2 and anti‐phospho‐serine/threonine (p‐Ser/Thr) antibodies. (B, D, F, H) The relative phosphorylation levels were quantified using imagej. Data were analysed using Student's *t*‐test and are presented as the mean ± SD of three independent experiments. ns, not significant. **P < 0.01 and ***P < 0.001.

To better understand the potential mechanisms of HMGA2 involved in cisplatin resistance, we transfected Flag‐HMGA2 plasmids into 293T cells, then treated cells with cisplatin. The phosphorylation status of HMGA2 was determined by immunoprecipitation and western blot assays with the specific antibody. As shown in Fig. 3A–D, cisplatin elevated the phosphorylation level of HMGA2 in a time‐ and dose‐dependent manner. Additionally, the endogenous phosphorylated HMGA2 was determined in HeLa cells. The data obtained indicated that HMGA2 phosphorylation was significantly increased upon stimulation of cisplatin (Fig. 3E–H). These data suggested that the enhancement of HMGA2 phosphorylation induced by cisplatin might contribute to the chemoresistance of cervical cancer cells.

### CSNK2A1 interacted with and phosphorylated HMGA2

**Fig. 4:**
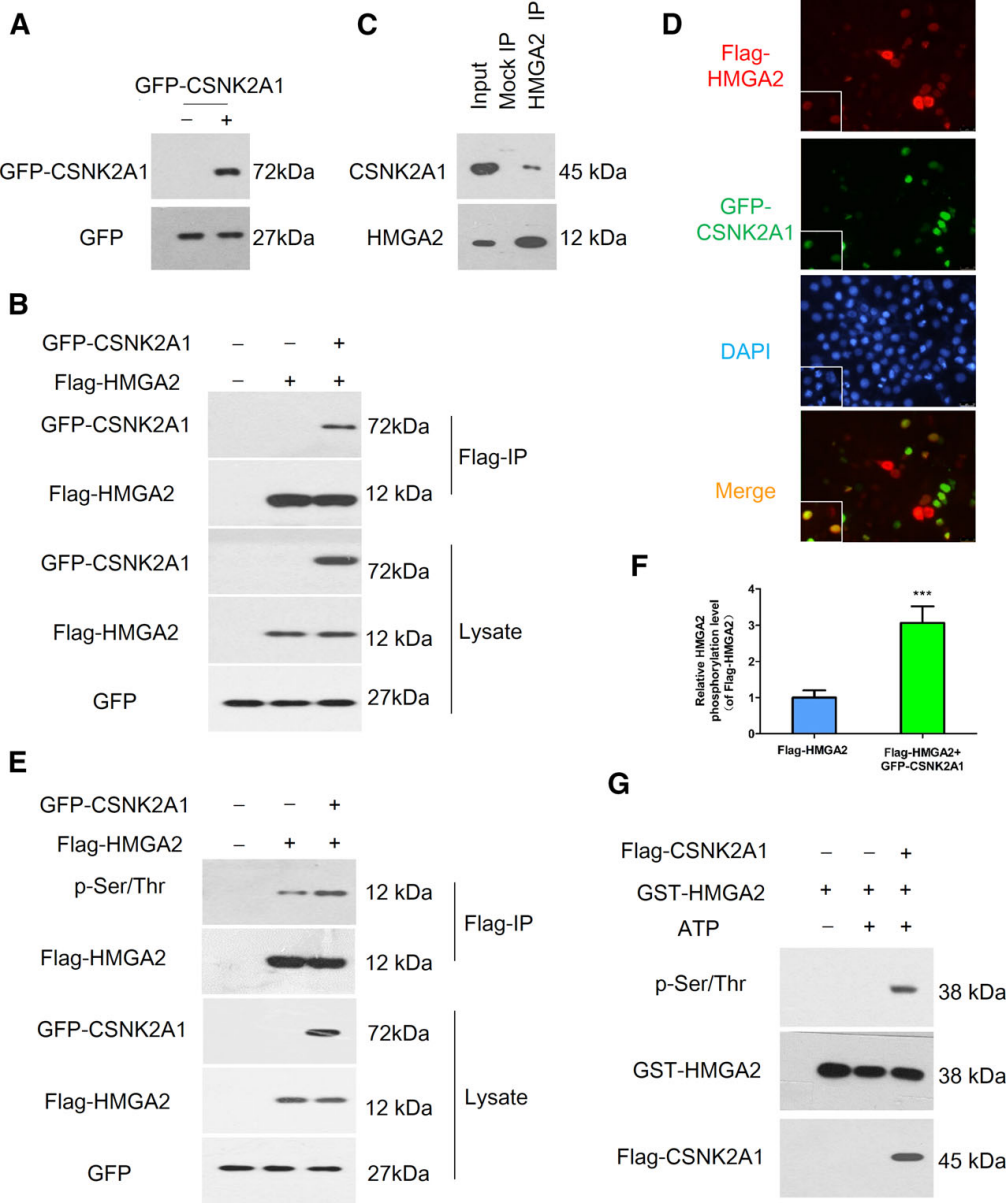
The relationship between HMGA2 and CSNK2A1. (A) Confirmation of the plasmid of GFP‐CSNK2A1 by a western blot assay. (B) 293T cells co‐transfected with indicated plasmids were lysed and immunoprecipitated using Flag‐M2 beads followed by a western blot assay with anti‐GFP and anti‐Flag antibodies. (C) An endogenous HMGA2‐CSNK2A2 complex was verified in HeLa cells. HeLa cells extracts were immunoprecipitated against HMGA2 or IgG isotype antibodies (Mock IP) and were analyzed by immunoblotting using anti‐CSNK2A1 antibody. (D) HeLa cells co‐transfected with Flag‐HMGA2 and GFP‐CSNK2A1 were stained for Flag (red) and GFP (green), visualized by fluorescence microscopy, and nuclei were stained with DAPI. (E) 293T cells transfected with indicated plasmids were lysed and immunoprecipitated using Flag‐M2 beads and a western blot assay was used for phosphorylation of HMGA2 by anti‐p‐Ser/Thr antibody. (F) Relative HMGA2 phosphorylation levels were quantified using imagej. (G) An *in vitro* phosphorylation assay showed that CSNK2A1 phosphorylated HMGA2. Data were analysed using Student's *t*‐test and are presented as the mean ± SD of three independent experiments. ***P < 0.001.

Based on the aforementioned results, we aimed to idenitfy the potential upstream kinase that was responsible for the phosphorylation of HMGA2. Numerous evidence has confirmed that CK2 had diverse functions as a protein kinase. We therefore assumed that CSNK2A1 was also involved in regulation of the HMGA2 phosphorylation. To determine the relationship between the two genes, we successfully constructed plasmids overexpressing CSNK2A1 (Fig. 4A). Subsequently, we transiently co‐transfected the plasmids of Flag‐HMGA2 and GFP‐CSNK2A1 or GFP‐vector into 293T cells, followed by immunoprecipitation with anti‐Flag M2‐agarose. The precipitated proteins were analyzed by a western blot assay using anti‐Flag or anti‐GFP antibodies. As shown in Fig. 4B, HMGA2 interacted with CSNK2A1. We then performed co‐immunoprecipitation assays of endogenous HMGA2 and CSNK2A1 proteins in HeLa cells. Cell extracts were immunoprecipitated against endogenous HMGA2 and the immunoblot for the CSNK2A1 antibody showed a clear signal for endogenous co‐immunoprecipitation between HMGA2 and CSNK2A1 (Fig. 4C). Using fluorescence microscopy, we observed that HMGA2 and CSNK2A1 co‐localized in the nuclei (Fig. 4D). Additionally, as shown in Fig. 4E,F, the phosphorylation level of HMGA2 was increased with the addition of CSNK2A1. To further investigate whether CSNK2A1 directly regulates HMGA2 phosphorylation, we performed an *in vitro* phosphorylation assay. The results demonstrated that CSNK2A1 phosphorylated HMGA2 *in vitro* (Fig. 4G). Therefore, these findings suggested that CSNK2A1 interacted and mediated the phosphorylation of HMGA2, which might be crucial for the function of HMGA2.

### HMGA2 regulated chemoresistance in a phosphorylation‐dependent manner

**Fig. 5:**
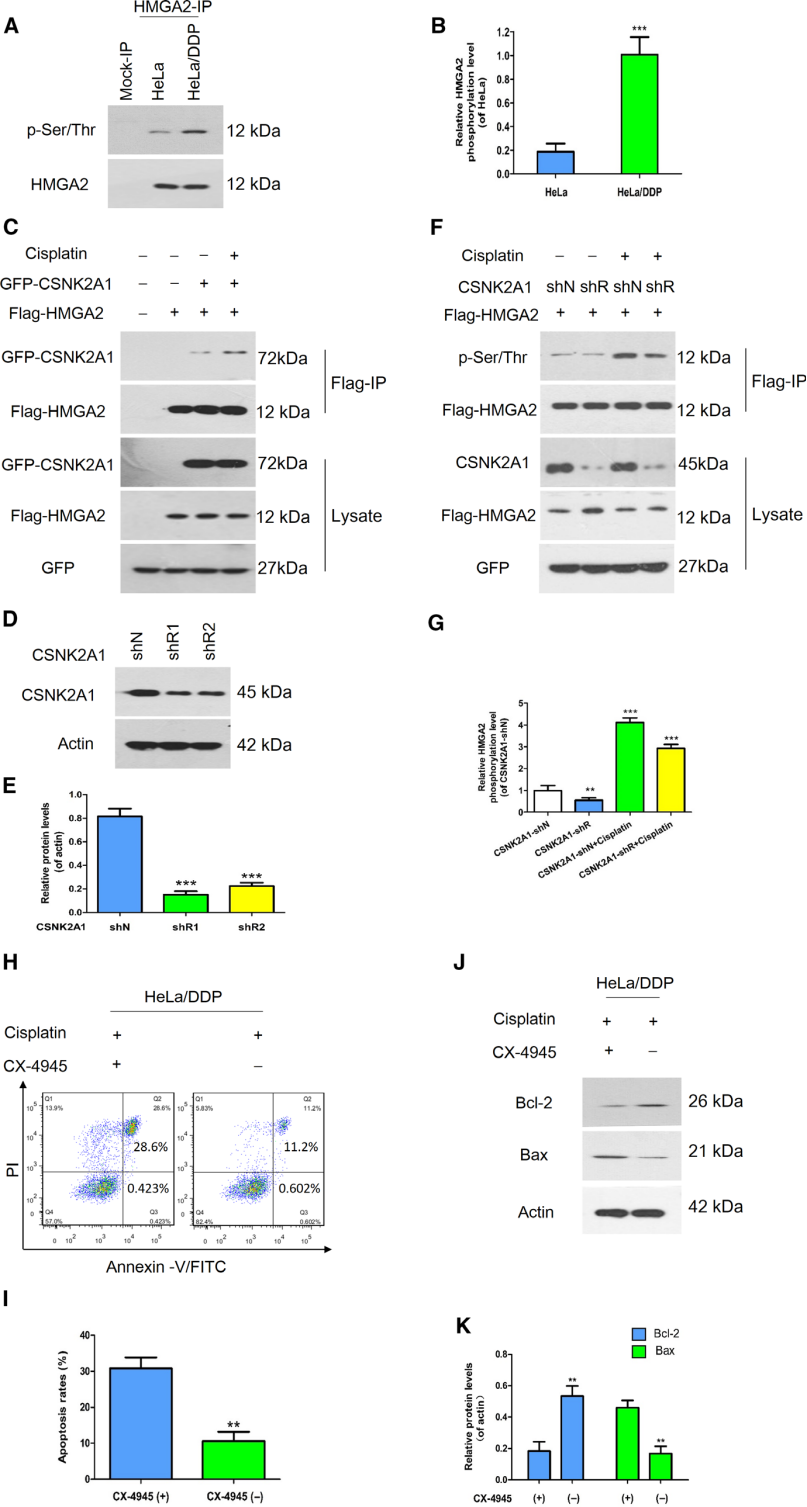
Effect of CSNK2A1 on HMGA2‐mediated chemoresistance. (A, B) The phosphorylation levels of HMGA2 were measured and quantified in cisplatin‐resistant HeLa cells and control cells. (C) 293T cells overexpressing the Flag‐HMGA2 with or without the plasmids of GFP‐CSNK2A1 were treated with cisplatin. Immunoprecipitation and western blot assays were performed with the indicated antibodies. (D, E) CSNK2A1 was knocked down in HeLa cells with shRNA. (F) HeLa‐CSNK2A1‐shN or HeLa‐CSNK2A1‐shR cells were transfected with Flag‐HMGA2 plasmids, and then treated with or without cisplatin at the indicated time point, followed by immunoprecipitation with Flag‐M2 beads. The precipitated proteins were immunoblotted with antibodies against Flag, CSNK2A1, GFP or p‐Ser/Thr. (G) The HMGA2 phosphorylation levels were quantified using imagej. (H, I) HeLa/DDP cells were treated with cisplatin+CX‐4945 (left) or cisplatin (right). Apoptotic cells were analyzed by flow cytometry. (J, K) The levels of apoptosis‐related proteins in HeLa/DDP cells were measured and analyzed by a western blot assay and imagej. Data were analysed using Student's *t*‐test and are presented as the mean ± SD of three independent experiments. ***P < 0.001.

To further confirm the relationship between HMGA2 and CSNK2A1 under cisplatin, we conducted immunoprecipitation and wblot assays. We first compared the phosphorylation levels of HMGA2 in cisplatin‐resistant cells (HeLa/DDP) with that in HeLa cells. The HMGA2 phosphorylation level was increased in HeLa/DDP cells (Fig. 5A,B). Next, we found that cisplatin enhanced the interaction between CSNK2A1 and HMGA2, which might be responsible for the up‐regulation of HMGA2 phosphorylation after treatment with cisplatin (Fig. 5C). Additionally, we constructed the CSNK2A1‐shRNA plasmids (Fig. 5D,E). As shown in Fig. 5F,G, the phosphorylation level of HMGA2 was suppressed with down‐regulation of CSNK2A1. To further support the findings observed in the CSNK2A1 knockdown cells, HeLa/DDP cells were treated with cisplatin, with or without CX‐4945, a potent and selective small molecule inhibitor of CK2, which exhibits strong anti‐tumor activity. Using a cell apoptosis assay, we found that the cell death rates were increased significantly in the combined treatment group compared to cisplatin treatment alone (Fig. 5H,I). As shown in Fig. 5J,K, after stimulation with CX‐4945, the Bcl‐2 protein levels in HeLa/DDP cells were significantly lower compared to that in the cisplatin‐only group, whereas the expression levels of Bax were increased in the combined‐treatment group. These results indicated that CSNK2A1‐mediated phosphorylation of HMGA2 was essential for enhancing cisplatin‐induced apoptosis.

## Discussion

The incidence of cervical cancer has decreased markedly in developed countries over recent decades, although it remains high in developing countries as a result of unhealthy lifestyles [24]. Drug resistance is one of the main causes of treatment failure with respect to cervical cancer and various studies have focused on this [25, 26, 27]. In the present study, we found that the phosphorylation level of HMGA2 was associated with the increased chemoresistance in cervical cancer cells. For the first time, the interaction of CSNK2A1 and HMGA2 was confirmed in cervical cancer. Cisplatin could increase the phosphorylation of HMGA2 by enhancing the interaction between CSNK2A1 and HMGA2.

As a high mobility group protein, HMGA2 has diverse functions in biological processes [28, 29]. In our previous study, we found that HMGA2 promoted the development and progression of prostate cancer by regulating epithelial–mesenchymal transition and matrix metalloproteinases [7]. In the present study, we found that HMGA2 was also involved in chemoresistance in cervical cancer, which was biologically consistent with our previous study. Furthermore, the present study confirmed the role of the interaction of HMGA2 and CSNK2A1 in cisplatin resistance for cervical cancer. Wang *et al*. [30] found that the interaction of CK2 and its substrate HMGA1 was associated with tyrosine kinase inhibitor resistance for EGFR‐mutant non‐small cell lung cancer and, in addition, HMGA1 and HMGA2 were found to have a very similar structure that includes three conserved domains [31], which supports our results indirectly. Previous studies have reported that the CK2 inhibitor, CX‐4945, exhibits antitumour efficacy, with synergistic effects in combination with chemotherapeutics [32, 33]. In the present study, we tested the effects of CX‐4945 in HeLa/DDP cells and the results showed that, after stimulation with CX‐4945, the cell death rates and the expression level of Bax were increased, whereas the expression level of Bcl‐2 was decreased. These data suggest that the combination including CX‐4945 sensitized cervical cancer cells to cisplatin, and this might partially occur via down‐regulation of HMGA2 phosphorylation.

Interestingly, as shown in Fig. S1, we found that there was no change in the expression level of HMGA2 after treatment with cisplatin. HMGA2 is chromatin architectural protein that does not have transcriptional activity itself but can modify chromatin structure by interacting with other kinase molecules [31]. Given our results described above, we inferred that the role of HMGA2 in cisplatin resistance was independent of the expression level but dependent on the interaction with protein kinase CSNK2A1.

The novelty of the present study is that we first demonstrated the role of the interaction of HMGA2 and CSNK2A1 in cisplatin resistance for cervical cancer. However, our study is limited by a lack of information regarding potential phosphorylation sites, for which additional investigations will be carried out in the near future.

In the present study, we first reported the phosphorylation of HMGA2 played an important role in cisplatin resistance for cervical cancer, which prompts a new direction for investigating the effects of HMGA2 in the future. Furthermore, we found that the kinase CSNK2A1 interacted and regulated the function of HMGA2 through its activated phosphorylation. In sum, we have demonstrated that the CANK2A1/HMGA2/Bcl‐2/Bax axis modulates the sensitivity of cervical cancer cells to cisplatin, and potentially provides new therapeutic targets for overcoming chemoresistance in cervical cancer.

## Conflict of interests

The authors declare that they have no conflicts of interest.

## Author contributions

SZ performed most of the experiments, and also drafted and revised both the figures and the manuscript. WD performed the cell culture. XH performed the cell proliferation detection. YJ re‐edited the manuscript and provided suggestions on the revision. SXQ supervised the project and modified the manuscript.

## Supporting information


**Fig. S1**. Effects of cisplatin on the expression of HMGA2. (A, B) HeLa cells were treated with or without cisplatin for 24 h, then a western blot assay was conducted to evaluate the expression of HMGA2. (C, D) HMGA2 expression were tested in the cisplatin resistant cells (HeLa/DDP). Data were analysed using Student's *t*‐test and are presented as the mean ± SD of three independent experiments. Ns, not significant.

## Data Availability

The analyzed data sets generated during the present study are available from the corresponding author upon reasonable request.
