# Supplementary figures and images for: CSNK2A1‐mediated phosphorylation of HMGA2 modulates cisplatin resistance in cervical cancer
Source: FEBS Open Bio. 2021 Jul 12;11(8):2245–55. doi: 10.1002/2211-5463.13228 (PMC8329774; doi:10.1002/2211-5463.13228)

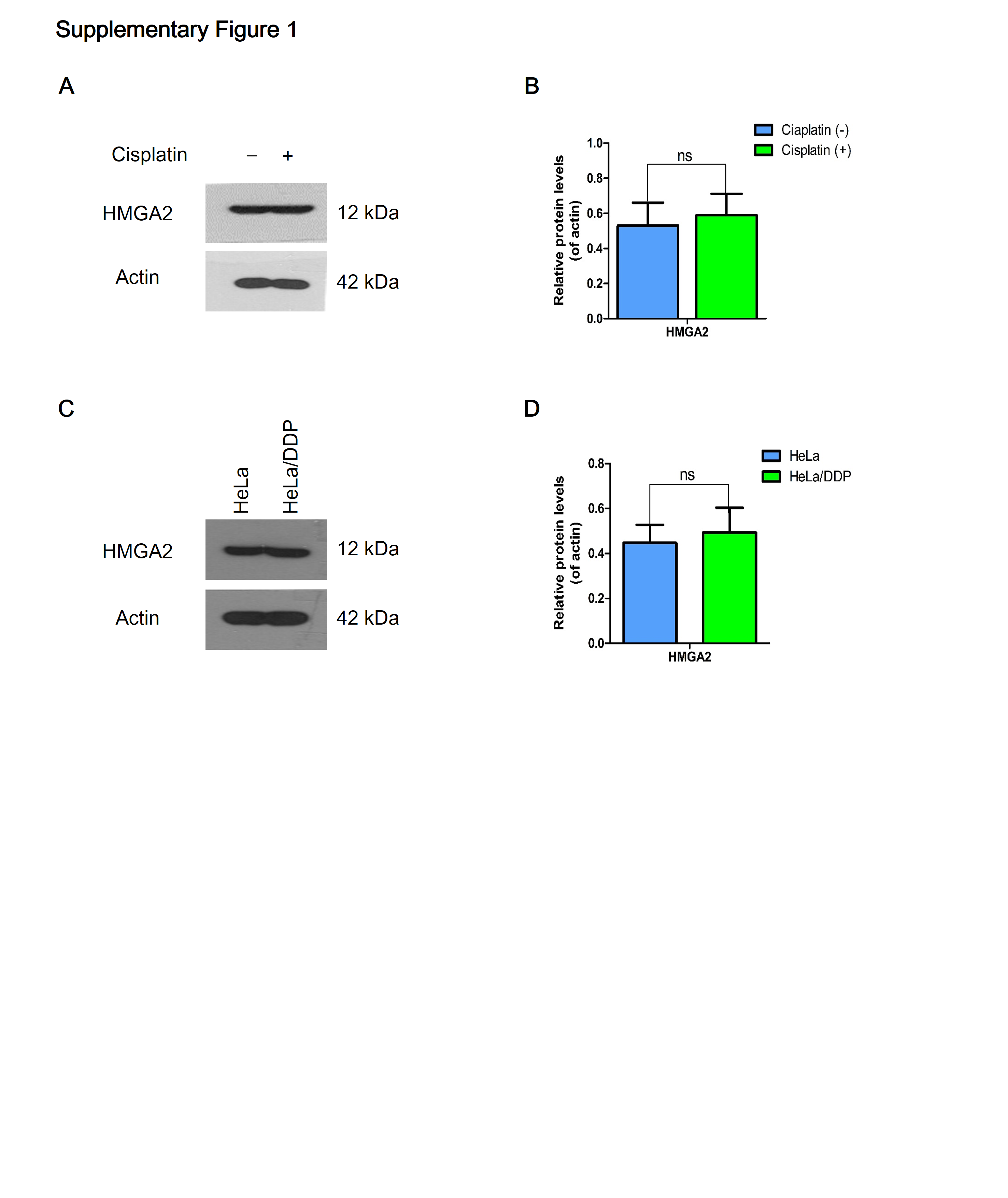

Supplement: Supplementary file 1 — Fig. S1. Effects of cisplatin on the expression of HMGA2. (A, B) HeLa cells were treated with or without cisplatin for 24 h, then a western blot assay was conducted to evaluate the expression of HMGA2. (C, D) HMGA2 expression were tested in the cisplatin resistant cells (HeLa/DDP). Data were analysed using Student's t‐test and are presented as the mean ± SD of three independent experiments. Ns, not significant. [file FEB4-11-2245-s001.TIF]
